# Acceptability of study procedures (self-collected introital swabs, blood draws and stool sample collection) by students 10–16 years for an HPV vaccine effectiveness study: a pilot study

**DOI:** 10.1186/s13104-016-1984-8

**Published:** 2016-03-16

**Authors:** Miriam Nakalembe, Twaha Mutyaba, Florence Mirembe

**Affiliations:** Department of Obstetrics and Gynecology, College of Health Sciences, Makerere University, Kampala, Uganda

**Keywords:** HPV vaccine, Acceptability, Self-collected vaginal swabs, Blood, Stool, Uganda

## Abstract

**Background:**

A cohort study was planned to evaluate vaccine immunogenicity and effect of malaria and helminth co-infections on the bivalent Human papilloma virus (HPV) vaccine. The study would involve self collected introital swabs, blood draws and stool sample collection. We therefore conducted a pilot study to assess the acceptability of these procedures among the students and their parents.

**Results:**

A cross-sectional study among forty four students from two purposively selected primary schools of Western Uganda. Exit interviews and two focus group discussions (FGD) (for parents) were conducted. Acceptability was measured by willingness to undergo the procedures again, recommending the procedures to others as well as proportion of introital swabs positive for β globulin. FGD determined acceptability of the parents and explored opinions and perceptions that would influence their decisions. HPV-16/18 and β globulin deoxyribonucleic acid (DNA) were analysed using a polymerase chain reaction (PCR) kit. All the students (100 %) in the study were willing to provide a self- collected introital swab and a stool sample as well as recommending their friends while (86.3 %) were willing for blood draws. There were 40/44 (90.1 %) self collected introital swabs that had positive result for human β globulin though none of them was positive for HPV-16/18. In the FGD, it emerged that parents concerns were on the blood draws and introital swab collection which were addressed.

**Conclusions:**

The study procedures were highly acceptable among this study population of students and their parents. Follow-up to assess HPV vaccine effectiveness and factors that may influence the vaccine in this age group is feasible.

**Electronic supplementary material:**

The online version of this article (doi:10.1186/s13104-016-1984-8) contains supplementary material, which is available to authorized users.

## Background

Cervical cancer is the second most common malignancy among women worldwide and the commonest cancer among—Ugandan women [1, 2]. Human papilloma Virus the commonest sexually acquired infection has been recognized as the necessary cause of cervical cancer [3].

The available vaccines against HPV infection are almost 100 % effective in preventing infection and high-grade pre- cancerous lesions associated with the HPV-16/18 included in the bivalent vaccine [4]. About 41 African countries have endorsed the use of the HPV vaccine. However, yet data on the effectiveness of the vaccine in Africa is still sparse yet prevalent immune compromising conditions such as malaria and helminths may affect the effectiveness of the vaccine [5].

In addition, the duration of protection and need for booster doses are not known, therefore, there is need for continued monitoring of vaccine immunogenicity and effectiveness over time [6].

A study was therefore conceptualized to evaluate vaccine immunogenicity and effect of malaria and helminth exposure on the vaccine in Uganda. This study would require multiple invasive and private procedures; blood draws, self-collected vaginal swab and a stool sample. Acceptability of self collected vaginal/introital swabs has been well documented [7]. However, preference for a physician collected sample as opposed to a self collected sample has been reported in a study among adolescents [8]. Additionally in Uganda, studies on self collected vaginal swabs have been conducted among older age groups [9, 10]. The main study was not only going to collect introital specimens but blood and stool samples as well. Therefore, to improve participation in the study and minimize loss to follow up, it was necessary to know whether the procedures that were to be used to collect the study samples were physically and culturally acceptable to this young study population and their parents. We therefore carried out a pilot study to assess acceptability of the study procedures that were to be used in the main study.

## Methods

### Study design

We conducted a cross section study in February 2011. Study participants were students aged 10–16 years from two Western Ugandan districts of Ibanda and Mbarara. HPV vaccination had taken place in Ibanda district and none in Mbarara. Ibanda district had about 272 primary schools while Mbarara district had 402 primary schools according to the district records. We purposively selected two neighboring schools one from each district that were near each other and near the road with at least 20 students in primary five. All the students from the two selected schools in primary level five were eligible to participate. The head teachers from these schools were contacted and detailed explanations about the study were given. Prior appointments were made for the teachers to request the parents/guardians of the students to come to the school. Presentations and discussions were held with the, parents/guardians as well as the students in separate sessions. These included information about the HPV vaccine; need to assess its effectiveness and the study procedures the students would undergo. Parents/guardians who consented and with their students’ assent were included in the survey. After the consent and assent, study numbers were assigned serially and a plaster bearing the number was placed on the left arm of the participating students. Students who were menstruating were excluded.

### Introital sample collection

A demonstration was given to the students on how to collect introital swabs by the principal investigator in the classroom. Students were instructed to wash their hands. In a squatting position with a Dacron swab the students were to open the swab by the end of the handle, rotate it around the introitus while counting up to 10 and then place it in a labeled sterile tube. With the above instructions, in the privacy of a restroom, the students self-collected an introital swab and returned them to the research assistants.

### Stool collection

The students were requested to place a stool sample of about a teaspoon on a polythene sheet bearing their unique number by use of a wooden spatula *from the rest room.* After collecting the stool, it was immediately examined by two skilled technologists using the Kato-Katz method [11] for presence of helminths.

### Blood sample collection

The study laboratory technician, carried out the blood draws from the students. In a sitting position, the girl’s non dominant hand was identified. Following identification of the vein in the cubital fossa, the area was cleaned with 70 % alcohol swab. Five millilitres (*mls*) of blood were aseptically collected from the cubital vein into a labelled serum separator tube (*SST*™) *tube*.

The vials of blood were put in transport boxes that had ice boxes and transported to Mbarara University Epicenter laboratory within 2–8 h of collection. The samples were then transported on dry ice to the Molecular laboratory at the Makerere University College of Health Sciences and kept at −80 °C.

### Isolation of HPV-16 and HPV-18 DNA

The dry swabs were soaked in 200 microliters (µl) of Tris Ethylenediaminetetraacetic Acid (EDTA) buffer for 2 min and vigorously vortexed to remove the swab material. The solution was transferred in a new tube and centrifuged and the supernatant was discarded. DNA was isolated from 100 µl of the remaining sample and HPV-16/18 genotyping was done by a commercial multiplex Real Time amplification according to the manufacturer’s instructions (Sacace Biotechnologies S.r.l. Via Scalabrini, 4422100 Como Italy on a Rotor Gene Q RG 6000 Gorbett Research). The PCR reaction involved primers for the human β globulin gene as an internal control. Samples that were negative for β globulin represented non compliance from the student to collect the sample.

#### Exit interviews

After the swab, blood and stool samples were collected we conducted exit interviews of all the students who had participated in the study were conducted to measure acceptability of the procedures by the students. The interviews were administered by use of an interviewer administered questionnaire. The students were asked whether they would undergo the same procedures in future as well as recommending the procedure to their friends. This lasted about 10–15 min (Additional file 1).

#### Focus group discussions

Two focus group discussions were held after the consent/assent process with 2 groups of six female and male parents of the participating students. The groups were picked purposively based on observed expressions of opinions during the consent/assent process. Half of each group comprised of those parents who had actively participated in the presentation session while the other half consisted of those who were relatively quiet. The focus group discussions were conducted in the local language and they lasted for about 1 h 30 min for both groups. The focus group guide used was based on a priori themes that included opinions and perceptions about each study procedure and what would influence their decision on having their daughters participate in the study in future. While we started with a priori themes, as the discussion progressed, more probing occurred based on emerging pertinent issues from the participants. The discussions were recorded and notes were taken by a research assistant who moderated the focus group discussions. The recorded information was translated as it was being transcribed into English as verbatim (Additional file 2).

### Data analysis

#### Quantitative data

Students who self reported sexual exposure and those who were positive for helminth were presented as proportions of the total number of students. Mean age and corresponding standard deviation were determined for the students using Epi Info™ 7 version. Acceptability of study procedures among the girls was determined by computing the proportions of those willing to undergo study procedures in future and those who would recommend other students for the procedures.

#### Qualitative data

The transcribed verbatim scripts and notes were read and reread by the principal investigator for familiarization with the content. This data was then coded for emerging themes based on thematic content analysis.

#### Ethical consideration

The pilot study was approved by the higher degrees research and ethics committee (HDREC) of the Makerere University College of Health Sciences and the Uganda National Council for Science and Technology (UNCST).

Permission was also sought from the local governments of the study districts, school authorities and parents/guardians of the students and finally from the students who participated in the study. Written informed consent was obtained from the parents/guardians of the students who participated in the study and a written signed assent was obtained from the individual students. Privacy and confidentiality were strictly observed participation in the study was voluntary. Students who were found with helminthes infection were treated according to the Uganda National guidelines. In addition, the parents/guardians who participated in the focus group discussions gave informed consent to participate in the study.

## Results

### Exit interviews with the girls

A total of 52 school students in grade 5 were approached to bring their parents to school; 45/52 (86.5 %) returned with their parents. The main reason for those that didn’t turn up was that they were attending to their gardens given the rainy season. Forty four 97.8 % students were enrolled while one girl was excluded due to menstruation.

Their socio-demographic characteristics are shown in Table 1. All were from rural schools with an age range of 10–16 years (mean 13.8 years; SD 1.67). Six out of 44(14 %) had ever had sexual exposure. None of the students was aware of their HIV status. A total of five students (11.3 %) were found to have helminths. Out of the 44 self collected vaginal swabs, 40 (90.1 %) samples were valid (β globin positive) on analysis. None of them was positive for HPV-16/18.

**Table 1:** Socio demographic and sexual characteristics of the respondents (Students) (n = 44)

Variable	Distribution (%)
Age (years)	
<12	5 (11.3)
≥12	39 (88.7)
Religion	
Catholic	24 (54.5)
Anglican	20 (45.5)
Sexual encounter	
Yes	6 (13.6)
No	38 (86.4)
Helminths	
Yes	5 (11.3)
No	39 (88.7)

The table shows the socio-demographics of the 44 students

Most of the students were above 12 years of age. About 6/44 (13 %) self reported initiating sexual activity. They were all of unknown HIV serostatus and about 5/44 (11.3 %) had evidence of helminths infection

All students were willing to undergo the procedure again if necessary, and would recommend other students for the procedures. This gave acceptability for self-collected introital swab and self-collected stool sample of 100 %. Of the 44 students, 38 (88.4 %) were willing to provide a blood sample in future. Those who were not willing to participate gave pain during blood draw as the reason.

### Focus group discussion with the parents

The 12 participants included parents with an age range of 27–45 years, all Christians and of the same local language and diploma as the highest education level. Three major themes emerged with relevance to acceptability of study procedures: (1) Fear- of imagined process, consequences of the procedures and other undisclosed possible use of sample; (2) favorable opinion of the stool exam; (3) willingness to support vaccination programs and see them succeed.

### Fear

#### Of consequences of the procedures

*‘Eh, how can my little girl provide all that blood, won’t this cause some of our children to collapse?’* (Mother of 11 year old girl).

The parents/guardians were concerned about the effect of blood draws on their daughters’ health and this feeling was shared by many of them. They thought that the 5 mls of blood collected from the students was too much and that it might lead to collapse. None of them knew how very little 5 mls was compared to the possible over 3000 ml which each student could have. With this clarity, this fear was dispelled.

#### Of imagined process

*Um, us the old women we go to Ibanda hospital and they put an instrument under…..too big for these small girls….you see they have not known these things of adults…hu ….I didn’t know how you people were going to do this I was wondering how this can happen……….after your explanation and what you have showed us they will use………..the little stick……outside only….eh…we are now happy that it will not be the same as for us’* (Mother of 13 year old girl).

The Program for Appropriate Technology in Health had set up cervical cancer screening centers and these women had the experience of cervical cancer screening which they had initially imagined would be the same procedure to be employed by the study. Though this fear was expressed here, these parents had already become comfortable with the procedure as it had earlier been demonstrated and they hoped it won’t change during the proposed study.

### Of other undisclosed possible use of samples

*‘Eh …eh….you people are just telling us other things for the blood…are you not going to use our childrens’ blood for other test like HIV ……um………..and others….we know some people want to study many things on Africans ………..’* (Father of 14 year old).

There was some suspicion that the blood collected would be used to test the students HIV status and may be other diseases. However, some of them held other views on this;

*‘Even if they study other things it is for our good…..our government protects us …um it is ok.*’ (Father of 11 year old).

The research team clarified on some aspects of ethics on research and they were reassured that they are protected by the principals of research ethics. This fear too was dispelled.

### Favorable opinion of the stool exam

*Eh …we are very happy about the stool exam………..you see our children can get treatment now if they are found with the worms…..that is very good……….. Thank you*. (Mother of 12 year old).

*“We were a bit uncomfortable that you were just taking our girls’ blood and not sure of the motives, but when we saw you checking stool as well, we are now sure you are thoroughly checking our girls, which is good”.* (Father of 15 year old).

All the parents were happy for their children to undergo the stool collection procedure and examination and expressed no problem with it many of them saying it was good for detection of worms.

#### Willingness to support vaccination programs and need for feedback

‘*We know vaccination has prevented diseases and we are happy….. we know our children will be well, but what about the results of the study’.* (Father of 12 year old student).

Majority of the parents were happy with the vaccination programs citing eradication of diseases like measles and polio. However many of them expressed need to know the results of the study for this vaccine that was given to their children.

*‘Um……. we see that our girls will be alright ….. when you come and explain everything to the parents and they understand……..our children can go ahead and participate in the study*. (Mother of 14 year old).

This view was echoed by majority of the parents and all of them expressed willingness to have their daughters participate in the future study.

## Discussion

Pilot studies are a crucial element of a good study design and can provide valuable insights into the design of the main study. Reasons for conducting them may include among others developing and testing adequacy of research instruments, assessing the feasibility of the main study in terms of recruitment approach, logistical issues (finances and staff), sample collection methods as well as training needs for the research team [12]. The general objective of the main study was to evaluate antibody responses elicited by the ASO4-adjuvanted HPV-16/18 vaccine and genital HPV infections among students in Western Uganda.

Therefore, we conducted the pilot study to mainly assess the acceptability of the procedures which were to be employed in the main study (blood draws, introital self-collected swabs and stool samples). In addition, during the conduct of the pilot study, valuable lessons were learnt which helped in the overall design of the main study.

There was total acceptability of self-collected introital swabs and self-collected stool samples. However, six students did not accept to have blood drawn from them because of fear of pain. The first two procedures (self-collected introital swabs and stool sample collection) were perceived to be painless while blood draw causes some transient pain. Despite the pain, a higher number would still undergo the procedure again. It is likely that this could improve even more if proper education and counseling are done on the necessity for the blood draw. The parents of the students expressed their fears about the procedures. They had these fears before they had the full explanations and demonstrations on how the procedures were to be done as well as the actual reason for the blood draw. After the demonstrations and explanations on the rationale of the exercise, the parents were able to understand the need for a study that will follow up the students to assess effectiveness of a vaccine that is hoped to help prevent cervical cancer a disease some of them had known about.

Stool exam was very well accepted as the parents quickly understood a direct benefit of having stool examination. The strength of these findings lies in the exit interviews and focus group discussions that were conducted so as to measure acceptability in this sample of students and their parents. Unlike in another study [13], these interviews were not based on expressing predetermined factors to the students. The students were able to determine for themselves whether or not the procedures had been acceptable, whether they would have it again or even recommend it to a friend. In the focus group discussions, the parents too were able to freely express themselves without being limited to predetermined responses on a questionnaire. The extensive explanations and demonstrations may have contributed to the high acceptability rates in our study. These results are in agreement with previous research findings which concluded that knowledge of the benefits and perception of risk are two most important factors in determining acceptability [13, 14]. Although these studies were vaccine acceptability studies, the principle of benefit and risk perception as far as predicting health related behavior cuts across and is well described in the Health belief model (HBM) which applies to this situation as well [15].

Despite the high acceptability, a small number of students may still refuse to undergo some of the procedures as evidenced in this study. Likewise, some parents may refuse to consent for their students. However, continuous education and sensitization to increase knowledge of benefits from HPV vaccine studies may enhance willingness to participate as evidence concerning hepatitis vaccine acceptability has showed [13].

HPV DNA samples may be clinician collected or self-collected [16]. The self-collected method is very suitable although in our study some few samples did not have evidence of human DNA on them which may imply none collection or sample inadequacy. The method eliminates use of a speculum which may not be appropriate for the age group both medically and culturally. The high acceptability of the self-collected introital swabs in our study is similar to other studies among adolescents and young women [10, 17]. In one of the studies [17], the adolescents found self testing for sexually transmitted diseases acceptable as it was private and less embarrassing. However, in one study involving adolescents and young women, clinician collected introital swab were preferred to self-collection [9]. This study somehow differed from ours and the other two studies above. It involved a much larger sample size with older students and was a hospital clinic based study while ours as well the other two studies were not hospital based; one was community based [10], while ours and the other one [17], were institution based. The participants had no complaints at all which factor may influence the preference of clinician over a self-collected sample.

On average, the students in this study were older than their counterparts in the same class in urban setting. This trend is common in Uganda where children in rural areas tend to start school later than their counterparts in the urban areas. On the other hand the rural students tend to start sexual activity earlier than urban students [18, 19]. This has implications on inclusion in HPV vaccine efficacy studies as these students are assumed not to have been exposed to the HPV virus just on the basis of their age. The vaccine is a prophylactic and inclusion of already infected students might distort the results.

The study had limitations. In our study, all the students were from two rural schools and were Christians. They were all from the same tribal region of Uganda. Given the tribal, cultural and religious diversity of Ugandans, the findings might not be generalisable to the rest of the Ugandan population. This is however from just a statistical view. We the researchers know that the study findings are more likely to apply to the rest of the Ugandan adolescent population since the fears that were expressed were not cultural or religious but physical for example pain.

However, despite some of these lessons, very useful lessons were learnt. The process of approach to the school administration, mobilization of the parents for consent at the schools was refined for the main study. Finding no evidence of DNA on some of the introital swabs led to the change from having the students collect the samples unsupervised to having supervised collection while maintaining privacy. The distance from the field to the Laboratory that was to finally store the sample before shipping was too far (300 kms) for daily deliveries of the samples. So after this pilot, we were able to identify a laboratory about 80 km away (longest distance). In this laboratory, we would be able to take samples daily for centrifuging and storage at −80° centigrade. We were also able to find ways of safely handling stool samples without contaminating the school compound.

## Conclusions

Proposed study procedures to assess the effectiveness of the bivalent HPV vaccines were highly acceptable to both parents and their daughters. Lessons learned from the pilot included collection of the introital swab under supervision, all helped to inform the design of the final study.

A follow up strategy to continue monitoring vaccine immunogenicity and effectiveness is therefore feasible in our setting and particularly in this group of young students.

## Additional files

10.1186/s13104-016-1984-8 Focus group discussion questionnaire: this includes the demographic details of participants, background information and introductory remarks as well as guiding questions for the discussions.
10.1186/s13104-016-1984-8 The pilot survey questionnaire: it includes the demographic characteristics of the girls who participated in the study as well as questions that assessed their willingness to participate in the study by providing blood, stool and self collected introital vaginal swab.

## Abbreviations

HPV: human papilloma virus
DNA: deoxyribonucleic acid
HDREC: Higher degrees research and ethics committee
UNCST: Uganda national council for science and technology
EDTA: ethylenediaminetetraacetic acid
SST*™*: serum separator tube
