# Supplementary material for: Acceptability of study procedures (self-collected introital swabs, blood draws and stool sample collection) by students 10–16 years for an HPV vaccine effectiveness study: a pilot study
Source: BMC Res Notes. 2016 Mar 16;9:170. doi: 10.1186/s13104-016-1984-8 (PMC4793529; doi:10.1186/s13104-016-1984-8)
Supplement: Supplementary file 2 — 10.1186/s13104-016-1984-8 The pilot survey questionnaire: it includes the demographic characteristics of the girls who participated in the study as well as questions that assessed their willingness to participate in the study by providing blood, stool and self collected introital vaginal swab. [file 13104_2016_1984_MOESM2_ESM.doc]

THE PILOT SURVEY -QUESTIONNAIRE

Questionnaire assessing the acceptability of the girls to participate in the study procedures for an HPV study.

| A: QUESTIONNAIRE IDENTIFICATION DATA | | | | | | | | | | | | | | |  |
| --- | --- | --- | --- | --- | --- | --- | --- | --- | --- | --- | --- | --- | --- | --- | --- |
| **STUDY 1D** |  | | | | | | | | | | | | | |  |
| LOCATION | | | | | | | | | | | | | | |  |
| A1. District | | 01=Ibanda | | | | | | 02=Mbarara | | | | | | |  |
| A2. Sub-county | |  | | | | | | | | | | | | |  |
| A3. School | |  | | | | | | | | | | | | |  |
| A4. School type | | 01= Day school | | | | | 02=Boarding school | | | | | | | |  |
| A5. Location setting of school | | 01= Rural | | | | | | | | 02=Urban | | | | | |
| A6. Village | |  | | | | | | | | | | | | | |
| A7. LCI Chairman | |  | | | | | | | | | | | | | |
| A8. Guardian’s name | |  | | | | | | | | | | | | | |
| A9. Telephone contact | | 1. | | | | | | | 2. | | | | | | |
| B. DEMOGRAPHIC DATA | | | | | | | | | | | | | | | |
| B1. Are you a boarder or day student | | 01=Day student. | | | | | | | 02= Boarding student | | | | | | |
| B2. What class are you in now? | | 01=P.5 | | | 02=P.6 | | | | | | | | 03=P.7 | | |
| B3. Date of Birth | | Day | | | Month | | | | | | | | Year | | |
| B4. Age in years | |  | | | | | | | | | | | | | |
| B5. How do you know your date of birth? | | 01= Told by guardian | | | 02= Just imagine | | | | | | | | 03= Evidenced by……… | | |
| C. VACCINATION | | | | | | | | | | | | | | | |
| C1. Vaccination status | | 01=Yes | | | | | | | 02=No | | | | | | |
| C2. Number of doses | | 01=1 | | | 02=2 | | | | | | | | 03=3 | | |
| C3. Evidence | | 01=Card | | | 02=Record | | | | | | | | 03= None seen | | |
| C4. Date of vaccination | | Day | | | Month | | | | | | | | Year | | |
| D. GENERAL MEDICAL HISTORY | | | | | | | | | | | | | | | |
| DI. TB treatment | | 01=No | | 02=Yes | | If no go to D3 | | | | | | | | | |
| D2. If yes when? | | Day | | | Month | | | | | | | | Year | | |
| D3. Heart disease | | 01=No | | | | | | | 02=Yes | | | | | | |
| D4. HIV status known | | 01=No | | | | | | | 02=Yes | | | | | 03=If yes, state | |
| **E. SEXUAL HISTORY** | | | | | | | | | | | | | | | |
| E1. Sexually active | | 01=No | | | | | | | 02=Yes | | | | | 03=If yes go to E2 and E3. | |
| E2. Number of sexual encounters | |  | | | | | | | | | | | | | |
| E3. History of condom use | | 01=No | | | | | | | 02= Yes | | | | | 03=If yes, go to E4 | |
| STUDY ID | | | | | | | | | | | | | | |  |
| Tell the girl that you are going to ask her some few questions concerning the study procedures they had undergone (self collected vaginal swabs, blood draw and stool). | | | | | | | | | | | | | | |  |
| F. STOOL EXAMINATION | | |  | | | | | | | |  | | | |  |
| F1. Are you willing to provide a stool sample to test for helminth again in future if requested? | | | 01= Very willing | | | | | | | | | 02= Willing | | |  |
|  |  |  | 03= Somehow willing | | | | | | | | | 04= Not willing | | |  |
|  |  |  | 05=Not sure | | | | | | | | | | | |  |
| F2. If no, please provide reasons for your answer. | | | 01= Don’t understand the reason for the test | | | | | | | | | 02= Am too young | | |  |
|  |  |  | 03= It was embarassing | | | | | | | | | | | |  |
| F3. Would you recommend this test to your friend? | | | 01= Yes, I would recommend | | | | | | | | | 02=No, I wouldn’t recommend | | |  |
|  |  |  | 03=Not sure | | | | | | | | |  | | |  |
| F4. If no, please provide reasons for your answer. | | | 01= I didn’t like it | | | | | | | | | 02=It was embarrassing | | |  |
|  |  |  | 03= It was not easy | | | | | | | | | | | |  |

| **STUDY ID** |  |  | |
| --- | --- | --- | --- |
| **G. HPV TESTING.** |  |  | |
| G1. Are you willing to provide a self collected vaginal swab again in future if requested | 01= Very willing | | 02= Willing |
|  | 03= Somehow willing | | 04= Not willing |
|  | 05=Not sure | | |
| G2. If no, please provide reasons for your answer. | 01= Don’t understand the reason for the test | | 02= Am a virgin and fear the stick will break virginity |
|  |  | | 04=I am not sexually active |
|  | 05= Other (specify)………………………… | | |
| G3. Would you recommend this test to your friend? | 01= Yes I would recommend the test | | 02=No, I would not recommend the test |
|  | 03= Not sure | | |
| G4. If no, please provide reasons for your answer. | 01= I didn’t like the test | | 02=It was painful |
|  | 03=It was embarassing | | |

| STUDY ID |  |  | |
| --- | --- | --- | --- |
| H. BLOOD DRAW |  |  | |
| H1. Are you willing to provide a blood sample again in future if requested | 01= Very willing | | 02= Willing |
|  | 03= Somehow willing | | 04= Not willing |
|  | 05=Not sure | | |
| H2. If no, please provide reasons for your answer. | 01= Don’t understand the reason for the test | | 02= The prick was painfull |
|  | 03= Don’t really have reason | | |
| H3. Would you recommend this test to your friend? | 01= Yes I would recommend the test | | 02=No, I would not recommend the test |
|  | 03= Not sure | |  |
| H4. If no, please provide reasons for your answer. | 01= I didn’t like the test | | 02=It was painful |
|  | 03=Not sure | | |

Interviewers name………………………………………

Date………………………………………………
